# Antioxidants in Cardiovascular Therapy: Panacea or False Hope?

**DOI:** 10.3389/fcvm.2015.00029

**Published:** 2015-07-06

**Authors:** Katarzyna Goszcz, Sherine J. Deakin, Garry G. Duthie, Derek Stewart, Stephen J. Leslie, Ian L. Megson

**Affiliations:** ^1^Department of Diabetes and Cardiovascular Science, Centre for Health Science, University of the Highlands and Islands, Inverness, UK; ^2^James Hutton Institute, Dundee, UK; ^3^Rowett Institute of Health and Nutrition, Aberdeen, UK; ^4^School of Life Sciences, Heriot Watt University, Edinburgh, UK; ^5^Cardiology Unit, Raigmore Hospital, Inverness, UK

**Keywords:** antioxidants, vitamin C, vitamin E, carotenoids, polyphenols, vascular disease, *N*-acetylcysteine, allopurinol

## Abstract

Oxidative stress is a key feature of the atherothrombotic process involved in the etiology of heart attacks, ischemic strokes, and peripheral arterial disease. It stands to reason that antioxidants represent a credible therapeutic option to prevent disease progression and thereby improve outcome, but despite positive findings from *in vitro* studies, clinical trials have failed to consistently show benefit. The aim of this review is to re-appraise the concept of antioxidants in the prevention and management of cardiovascular disease. In particular, the review will explore the reasons behind failed antioxidant strategies with vitamin supplements and will evaluate how flavonoids might improve cardiovascular function despite bioavailability that is not sufficiently high to directly influence antioxidant capacity. As well as reaching conclusions relating to those antioxidant strategies that might hold merit, the major myths, limitations, and pitfalls associated with this research field are explored.

## Introduction

Inappropriate oxidation of biomolecules is a hazard associated with all aerobic life. Harmful oxidation is often mediated by reactive oxygen species (ROS) that are generated by a wide range of biological processes, including mitochondrial respiration and both enzymatic and non-enzymatic chemical reactions. The long-standing but simplistic view of ROS is that they are harmful to cells, contributing to the aging process [the so-called free radical theory of aging (1)] and implicit in a wide range of disease processes, including cancer and cardiovascular disease. However, the complexity of the interaction between cells and ROS is evident in the fact that cells can generate ROS deliberately in small amounts to act as signaling molecules (2), deliberately in large amounts to act as part of the immune defense mechanism (3), or inadvertently through the respiratory chain (4) or other metabolic processes. Indeed, the free radical theory of aging is no longer universally accepted on account of the fact that two of the most effective interventions for improving health, caloric restriction and physical activity, induce a counter-intuitive induction of mitochondria-derived ROS (5). How then are the ROS generated under these conditions beneficial? The answer lies in the adaptive response that the cells mount to counter the potential for deleterious effects. In particular, there is strong evidence that repeated low-level bouts of oxidative stress up-regulate endogenous antioxidant defense mechanisms, which might drive paradoxical improved health and longevity (6). To label oxidative stress as universally harmful is, therefore, inaccurate; its propensity toward harm is a function of the site and amount of ROS generation and can be ameliorated or entirely reversed by the compensatory adaptive response.

Antioxidants are substances that “neutralize” ROS before they are able to react with cellular components and alter their structure or function. According to the free radical theory of aging, antioxidants were considered to be universally protective and beneficial; hence, the familiar manufacturers’ claims that foods, drinks, and supplements are “high in antioxidants,” with the subliminal inference that they must therefore be “good for you.” This generalization is misleading for a number of reasons. First, it implies that all antioxidants are the same; the truth is that the term “antioxidant” applies to a very wide variety of chemical entities that share only the capability of chemical reduction (donation of electrons). Importantly, dietary antioxidants span the chemical spectrum; some are highly lipophilic with long alkyl chains (e.g., vitamin E, carotenoids), while others are highly water-soluble (e.g., vitamin C). In addition, the size and complexity of antioxidants varies from the small and simple (e.g., salicylates; RMM ~170) to very complex polyphenolic agents, like tannic acid (RMM ~1700). This chemical diversity impacts heavily on bioavailability, metabolism, and cellular distribution of any given antioxidant; lipophilicity will predispose to accumulation in cell membranes and circulating lipoproteins, while water-soluble agents are unlikely to penetrate cell membranes without the aid of transporters. Bioavailability is a highly complex issue that depends on resistance to digestion and metabolic conversion by the gut microbiome (7, 8), absorption, metabolism, and clearance. Finally, antioxidants are, by definition, rapidly oxidized; while this is less of an issue for those that can be stored in solid form, it is an important consideration upon dissolution and in oils. Oxidation before or during ingestion might not only render an antioxidant inert but could also actively promote oxidative stress, depending on the nature of the product formed.

There is a wealth of literature relating to the benefits or otherwise of antioxidants in cardiovascular disease, but there is no consensus as to the relative merits of this therapeutic approach, partly on account of the heterogeneity among study populations, coupled with the wide variety of different antioxidant approaches undertaken – from dietary interventions (e.g., fruit and vegetables, foods, snacks, drinks, and multivitamins) to specific antioxidants (e.g., vitamin C, vitamin E, quercetin, resveratrol, epicatechin, *N*-acetylcysteine, allopurinol). While several clinical studies have suggested that diets rich in fruit and vegetables protect against cardiovascular disease (9–13), the evidence supporting the notion of protective effects of particular diets, or components therein, is both complex and contradictory. In this review, we aim to critically assess the preclinical and clinical evidence with a view to making some sense of the conflicting data. In particular, we will focus on the role of oxidative stress in atherogenic disease progression, prior to evaluating the evidence surrounding antioxidants in foods, drinks, and supplements.

## Oxidative Stress, Antioxidants, and Cardiovascular Disease

Oxidative stress – an imbalance between pro-oxidants and antioxidants, in favor of the former – is an important contributory factor to the atherogenic process (14). Many conditions linked to cardiovascular disease are associated with excessive pro-oxidant production and/or depression of endogenous antioxidant status; examples include diabetes, hyperlipidemia, hypertension, and obesity (15–19). In addition, a range of environmental factors [e.g., tobacco smoke (20–22), pollution (23, 24)] is known to contribute to oxidative stress and to promote cardiovascular disease. Pro-oxidant substances can be described as either free radical species or non-radical species that mediate peroxidation; the two major sub-groups are ROS and reactive nitrogen species (RNS; Table 1).

**Table 1:** **Pro-oxidant substances**.

Free radicals		Non-radicals	
Reactive oxygen species	Reactive nitrogen species	Reactive oxygen species	Reactive nitrogen species
Superoxide	Nitric oxide	Hydrogen peroxide	Peroxynitrite
Hydroxyl	Nitrogen dioxide		Nitrite
Hydroperoxyl			
Peroxyl			

Antioxidants are either endogenous, derived from diet, or in the form of therapies. Endogenous antioxidants include superoxide dismutase (SOD), catalase, glutathione (GSH), GSH peroxidase (GPx), thioreductase, and uric acid. Dietary sources include vitamins A, C, and E, as well as polyphenolic compounds and minerals (25), while *N*-acetylcysteine and allopurinol are therapeutic agents under investigation for antioxidant effects.

Coronary artery disease, stroke, peripheral vascular disease, hypertension, and heart failure are examples of cardiovascular diseases that are considered to be valid targets for antioxidant therapy. Atherosclerosis is the pathological process that underlies coronary artery disease, leading to myocardial infarction, as well as other vascular disease leading stroke and peripheral vascular disease. Given the importance of atherosclerosis in cardiovascular disease, it will form the focus of this review, although some of the studies described transgress the boundaries into related conditions, such as hypertension and heart failure.

## Atherosclerosis

Dysfunction of the endothelial cells that line all blood vessels is a key early event in the development of atherogenesis (26). The endothelium is an active monolayer of cells with a wide variety of key roles, including control of vascular tone, smooth muscle cell proliferation, platelet aggregation, inflammation, and maintenance of vessel wall permeability (14, 26, 27). Endothelial cells are particularly susceptible to oxidative stress, not only through ROS-mediated cell death but also because the bioavailability of the normally protective mediator, nitric oxide (NO), is compromised through its very rapid reaction with superoxide anion (28). As well as eliminating the protective effects of NO, this reaction generates peroxynitrite (ONOO^-^), a highly cytotoxic RNS, which can also mediate peroxidation of arachidonic acid derivatives to form isoprostanes and malondialdehyde (MDA), lipoproteins to form oxidized LDL, and *n*-6 polyunsaturated acids to form 4-hydroxynonenal (29–32).

**Figure 1:**
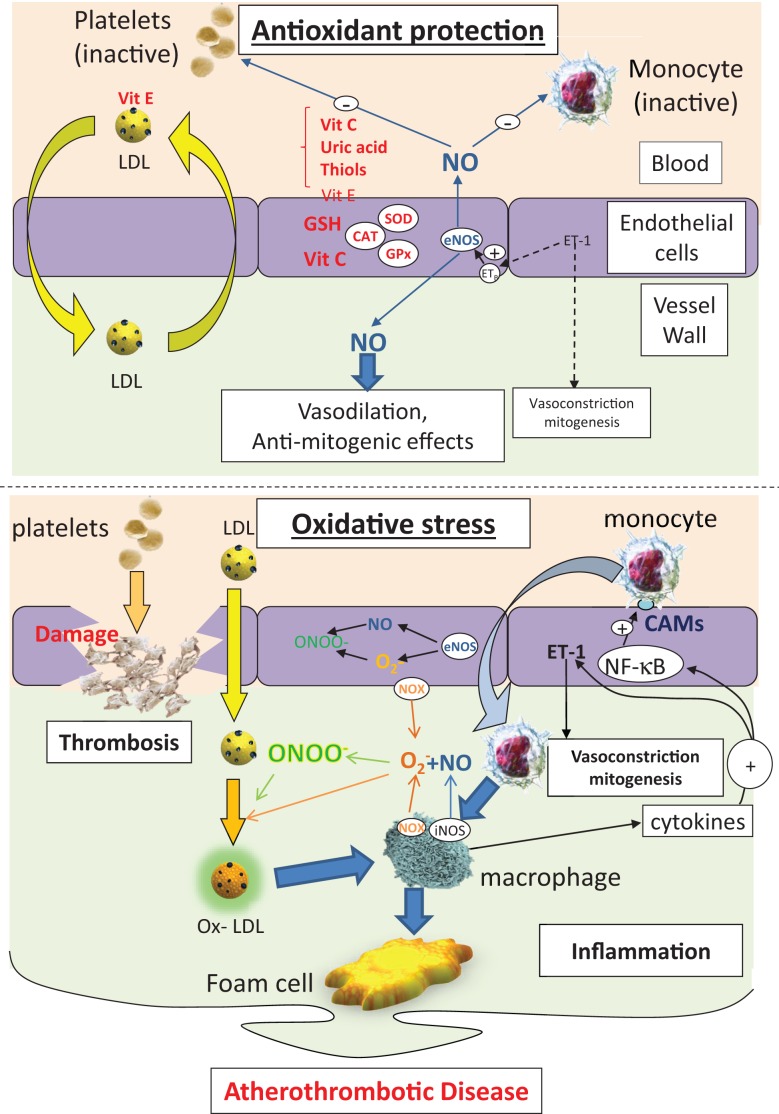
**The protective effects of antioxidants on endothelial function, LDL handling, inflammation, and thrombosis (top panel)**. Under conditions of oxidative stress, endothelial cell damage, loss of the protective effects of NO, and enhanced LDL peroxidation combines to drive an inflammatory state, leading to lipid accumulation in the arterial wall. CAMs, cell adhesion molecules; NOX, NAD(P)H oxidase; eNOS, endothelial NO synthase; iNOS, indicible NO synthase; NF-κB, nuclear factor kB; SOD, superoxide dismutase; ET-1, endothelin-1; CAT, catalase; GSH, glutathione; GPx, GSH peroxidase; LDL, low-density lipoprotein; ox-LDL, oxidized low density lipoprotein; ONOO^−^, peroxynitrite; ${\text{O}}_{2}^{\cdot \;-}$, superoxide; NO, nitric oxide.

Smoking (33), diesel particulate matter (23), diabetes (34), hyperlipidemia (35), and hypertension (36) are risk factors for atherosclerosis that are associated with oxidative stress. Disease progression is often considered to be an inflammatory process, which ultimately results in lipid deposition in the intima of the blood vessel wall (27, 37, 38). Atherosclerosis is a continuous process, but is often categorized into three stages: fatty streak formation, fibrous plaque development, and establishment of a complicated plaque. The fatty streak is instigated by damage to the endothelium, resulting in expression of cellular adhesion molecules, such as vascular adhesion molecule-1 (VCAM-1), which ultimately leads to recruitment of monocytes into the sub-endothelial space. Monocytes are activated by cytokines to differentiate into macrophages (Figure 1). Accumulation of macrophages in the vessel wall does not necessarily constitute a problem because, ordinarily, inflammation is rapidly resolved. However, a crucial step in progression of this inflammatory event into a chronic disease process hinges on oxidative stress because LDL, which usually diffuses freely across the endothelium, readily undergoes peroxidation to ox-LDL, triggering recognition by scavenger receptors on activated macrophages now resident in the vessel wall. Phagocytosis of ox-LDL by macrophages traps the lipoprotein in the intima and the now bloated macrophages take on a new identity – “foam cells” (39) (Figure 1). The fibrous plaque is characterized by a stable cap of proliferated smooth muscle cells and fibroblasts, which secrete collagen and other connective tissue, enveloping a cholesterol-rich, lipid, and collagen core. The final stage of plaque development, the complicated plaque, results in an unstable lesion characterized by inflammation, necrosis, ulceration, hemorrhage, and thrombus, which can ultimately be responsible for the occlusion of the blood vessel, with clinical consequences of myocardial infarction, stroke, or peripheral ischemia, depending on the location (40).

Oxidative stress has a clear role in the onset and the progression of atherosclerosis, impinging on a number of points in the disease process (Figure 1). As such, it is an attractive target for antioxidant therapy, but the success or otherwise of proposed interventions will rely on the ability to deliver sufficient amounts of the appropriate antioxidant to the right target, which is not a trivial issue to resolve.

## Antioxidant Defense

Cells synthesize and accumulate a wide variety of powerful antioxidants, including vitamins (A, C, and E), and enzymes (e.g., SOD, catalase, GPx, and thioredoxin reductase). Among other non-enzymatic antioxidants available for cells are GSH (41, 42), and the diet-derived free radical scavengers, carotenoids, and polyphenols, assuming that they accumulate in sufficiently high concentrations to be effective in this mode.

### Primary defense mechanisms

In a state of oxidative stress, antioxidants help to mitigate against damage by removing potential oxidants or transforming them into less reactive compounds. The function of the so-called primary defense mechanism is to prevent the oxidative damage directly by intercepting free radicals before they can damage intracellular targets. The endogenous enzymes are central in primary defense (43, 44). SOD is responsible for converting superoxide radical to hydrogen peroxide as follows:

$${{\text{O}}_{\text{2}}}^{.-}+{{\text{O}}_{\text{2}}}^{.-}+\text{2}{\text{H}}^{+}\;\frac{\text{SOD}}{\to }\;{\text{H}}_{\text{2}}{\text{O}}_{\text{2}}{+}^{}{\text{O}}_{\text{2}}$$

Primary defense against H_2_O_2_ is mediated by the enzymes catalase and GPx, which transform H_2_O_2_ into water and molecular oxygen:

$$2{\text{H}}_{2}{\text{O}}_{2}\;\frac{\text{catalase}}{\to }2{\text{H}}_{2}\text{O}+{\text{O}}_{2}$$

The GSH system is an important cellular defense mechanism against free radicals. GSH not only acts as a direct ROS scavenger but also plays a fundamental role in the regulation of the intracellular redox state. The system consists of GSH, GPx, and GSH reductase. GPx is an enzyme that catalyzes the reduction of H_2_O_2_ to water utilizing GSH as a co-substrate:

$${\text{H}}_{\text{2}}{\text{O}}_{\text{2}}+\text{2GSH}\;\frac{\text{GPx}}{\to }\;\text{GSSG}+\text{ 2}{\text{H}}_{\text{2}}\text{O}$$

Glutathione disulfide (GSSG) is then reduced back to GSH by GSH reductase:

$$\text{GSSG}+\text{ NADPH}+{\text{H}}^{+}\to \text{2GSH}+\text{ NAD}{\text{P}}^{+}.$$

The ability of cells to regenerate GSH (through the reduction of GSSG or by *de novo* synthesis of GSH) reflects efficiency of the cell in managing oxidative stress (44).

### Secondary defense mechanisms

Secondary antioxidant defense mechanisms – also known as chain-breaking defenses – trap harmful radicals before they can inflict damage. Many small molecules widely distributed in biological systems can scavenge free radicals as a part of the secondary defense system, including vitamin C, uric acid, and free and protein-incorporated cysteine (e.g., Cys 34 in albumin).

Lipid soluble antioxidants, such as vitamin E, are present in cell membranes and protect against lipid peroxidation. Vitamin E converts oxygen-centered free radicals to less reactive form by donating a hydrogen ion. Nuclear enzymes involved in DNA repair can be considered to be secondary defense systems against oxidative injury caused by oxygen free radicals. Many GSH transferases that express peroxidase activity are considered to also protect against lipid peroxidation (43).

## Antioxidant Therapies

In an effort to make some sense of the range of outcomes achieved with antioxidants and to take into consideration the concept that each antioxidant has different merits and should be considered as a separate entity, we will examine each of the most studied antioxidants individually. However, it is important to highlight the findings of large multivitamin supplement clinical trials to frame the findings for individual antioxidants (vitamin, mineral, or other) to follow. For clarity, we have not included supplements and drugs that have well-characterized primary actions that might be complemented by antioxidant activity. For example, antioxidant activity has been described as one of several pleiotropic effects attributable to statins (45–47), but their principle activity is through inhibition of hydroxy-3-methyl-glutaryl-CoA (HMG-CoA) reductase. Similarly, polyunsaturated fatty acids [e.g., omega-3 fatty acids, docosahexanoic acid (DHA) and eicosapentaenoic acid (EPA)] might have antioxidant effects (48–51), but they also have a major impact on the balance between pro- and anti-inflammatory eicosanoids (52–55). In these cases, more than others, it is difficult to attribute any effects seen in clinical studies to antioxidant activity as opposed to alternative actions, but it is nevertheless acknowledged that they have the potential for antioxidant effects.

### Multivitamin clinical trials

The blanket approach of daily multivitamin therapy is appealing because it negates the need to identify the active ingredient – simply deliver all the essential vitamins at the agreed recommended daily allowance to ensure that none are deficient. Another potential benefit of a multivitamin approach is that there might be synergistic effects of the vitamins and minerals included in the supplement. The risk, however, is that individuals might view multivitamin supplements as a substitute for a healthy fruit and vegetable-rich diet, leading to deprivation of other beneficial micronutrients and health-enhancing components (e.g., fiber), which might hold just as much, if not more, promise than the vitamins themselves.

A range of case–control studies have reported on the potential benefits of multivitamins in cardiovascular disease. While some show a reduction in cardiovascular risk associated with multivitamin use (56, 57), others do not (58–60). Perhaps most tellingly, a large (14,641 male participants) randomized placebo-controlled trial among US physicians (PHS II) failed to identify any difference in cardiovascular outcomes between the multivitamin group and the placebo group (61). A limitation of this study is that the study group (US physicians) is highly educated and likely to be well-nourished, with a balanced diet; vitamin supplements might be superfluous in such a group. Nevertheless, in response to these and other trials, the US Preventative Services Task Force statement regarding multivitamins for prevention of cardiovascular disease and cancer reads “no recommendation” on account of insufficient evidence to determine the balance of benefits and harm (62). Furthermore, a comprehensive Cochrane review (63) of the impact of antioxidant supplements (administered singly or in various combinations) on healthy individuals (26 trials), or in people with one or more of a range of diseases (52 trials), including cardiovascular disease (10 trials), found no overall reduction in all-cause mortality, irrespective of the combination of antioxidants used. Indeed, the findings of the review indicate that some antioxidants (β-carotene, vitamin A, and vitamin E) have the potential to increase mortality, either singly or in combination with other antioxidant supplements.

These findings and the conclusions drawn provide a backdrop for all of the data relating to individual vitamins and minerals that have been advocated in protection against cardiovascular disease, the essence of which is covered in the sections to follow.

### Vitamin C

#### Biochemistry

Vitamin C (ascorbate or ascorbic acid) is a water-soluble vitamin found in high concentrations in fruit and vegetables, particularly citrus fruit, kiwi, cantaloupe, mango, strawberries, and peppers (64). Dietary ascorbate is essential for humans because we lack the enzyme, l-gulono-γ-lactone oxidase, required to synthesize it (65). The recommended daily allowance for ascorbate varies from country to country (40–90 mg/day) derived from knowledge about both the minimum requirement to prevent scurvy – the debilitating disease that is caused by chronic vitamin C deficiency (66) – and the threshold plasma concentration that drives excretion. However, it has long been recognized that vitamin C is an antioxidant and that its consumption may be beneficial in reducing the impact of oxidative stress in a range of disease processes (64).

**Figure 2:**
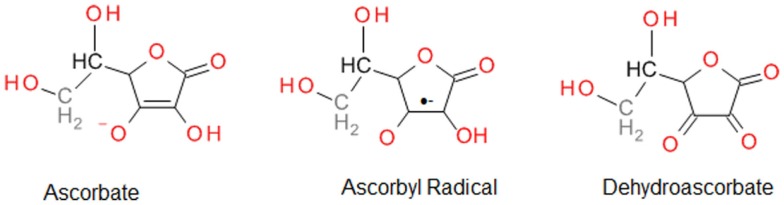
**Vitamin C (ascorbate) and its oxidation products**.

Vitamin C is a strong reducing agent that is reported to provide cytoprotection by scavenging ROS, and hence, protecting DNA, protein, and lipids against peroxidation (64). The powerful ability of vitamin C to eliminate ROS results in its oxidation to inactive dehydroascorbate; hence, its notoriety as a “sacrificial” antioxidant. However, it is important to recognize that vitamin C is also capable of recycling other endogenous antioxidants, including vitamin E (67). The free radical scavenging ability of vitamin C is attributed to donation of two electrons from a double bond between the second and third carbons in the ring (Figure 2). Among the ROS scavenged are superoxide, hydroxyl radicals, peroxyl radicals, NO, and many non-radicals, such as hydrochlorous acid and nitrosating agents (64).

The importance of vitamin C in cellular function is highlighted by its very high intracellular concentration (>1 mM) (68), which is considerably greater than that in the plasma (typically 30–80 μM). There are specific transport systems for uptake and accumulation of vitamin C in cells: sodium-dependent vitamin C transporters (SDVC-1 and SDVC-2) transport the reduced form of the vitamin, while the hexone transporters, GLUT-1 and GLUT-3, transport dehydroascorbic acid (69–71).

On a cautionary note, however, despite the many beneficial properties of vitamin C, in the presence of metal ions, it can be pro-oxidant by catalyzing the reduction of Fe^3+^ to Fe^2+^ (66), which can then take part in Fenton chemistry, producing highly reactive hydroxyl radicals, resulting in oxidative damage to proteins, lipids, and DNA (72):

$$\begin{array}{l} \text{F}{\text{e}}^{3+}{+\;}^{•}{\text{O}}_{{\text{2}}^{\text{-}}}\to \text{F}{\text{e}}^{2+}+{\text{O}}_{\text{2}}\\ \text{F}{\text{e}}^{2+}+{\text{H}}_{\text{2}}{\text{O}}_{\text{2}}\to \text{F}{\text{e}}^{3+}+\text{O}{\text{H}}^{-}{+\;}^{•}\text{OH} \end{array}$$

It is also important to acknowledge that, by definition, vitamins are only required in small quantities. Indeed, absorption of vitamin C occurs at variable rates, depending on the intake (~100% with low intake and as low as ~30% with high intake) (68). Similarly, there is relatively tight regulation of the plasma concentration on account of a renal re-absorption threshold for clearance at ~80 μM (68). In addition, vitamin C is readily oxidized by the endogenous enzyme, l-ascorbate oxidase; taken together, these biological regulators ensure a degree of homeostasis for plasma vitamin C at concentrations that rarely drift below 30 or above 80 μM. This is important because increased ingestion in an individual with a relatively healthy diet will have only a transient impact on plasma concentration and quite possibly none at all. Indeed, the recommended daily allowance has been reached with consideration of the pharmacokinetics of vitamin C.

#### *In Vitro* and *In Vivo* Animal Studies

The inflammatory response seen in atherosclerosis can be inhibited by vitamin C by preventing leukocyte aggregation and adhesion to the endothelium, induced by cigarette smoke *in vitro* (73). Vitamin C also has the potential to prevent lipid peroxidation, central to atherosclerosis, by inhibiting oxidation of LDL and subsequent uptake of ox-LDL (64, 74).

Mice lacking l-gulono-γ-lactone oxidase (Gulo^−/−^), the enzyme essential for vitamin C synthesis, show extensive vascular damage, including elastic lamina disruption, smooth muscle cell proliferation, and desquamation of endothelial cells, when vitamin C is withdrawn from their diets, highlighting the essential nature of vitamin C in vascular development and function (75). In the Apo-E^−/−^/Gulo^−/−^ atherosclerotic mouse model, plaque development was found to be unaffected by vitamin C deficiency, but reduced collagen deposition in plaques suggested the potential for increased risk of rupture in this *in vivo* animal study (76).

#### Clinical Studies and Trials

The fact that the majority of animal species synthesize vitamin C makes animal studies difficult. A number of small-scale clinical studies have investigated the impact of vitamin C on several factors associated with vascular health. The British Regional Heart Study found an inverse association between plasma vitamin C concentration and markers of inflammation and endothelial dysfunction in men with no history of cardiovascular disease or diabetes (77). In addition, acute administration of vitamin C (3 g) improved endothelium-dependent vasodilation in the epicardial coronary artery in patients with hypertension (78). Endothelial dysfunction has been identified as the major target for vitamin C-mediated effects. For example, in patients with coronary artery disease or hyperglycemia-induced impairment of vasodilation, NO-mediated vasodilation is restored with either oral (6 g over 2 days) or intra-arterial infusion (24 mg/min for 10 min) of vitamin C (79, 80). The precise mechanism underlying this effect is not yet clear, but it has been postulated that, as well as scavenging ROS, vitamin C might impact on NO bioavailability through direct stimulation of the enzyme responsible for synthesis of NO (endothelial NO synthase: eNOS), or enhanced synthesis of an essential cofactor for NOS activity, tetrahydrobiopterin (BH_4_) (81, 82). Likewise, oral vitamin C administration (2 g) was found to reduce arterial stiffness and platelet aggregation in healthy individuals; the mechanism involved was not established in this study, but improved endothelial function was considered to be a strong possibility (83).

The European prospective investigation into cancer and nutrition (EPIC) Norfolk study found that plasma vitamin C concentration was inversely related to incidence of cardiovascular disease-related mortality, as well as all-cause mortality, in both men and women. The study recruited nearly 20,000 individuals aged 45–79 years and monitored plasma vitamin C and all-cause mortality, with a particular focus on cardiovascular disease and cancer, for ~4 years. It was determined that by increasing plasma vitamin C by 20 μM through increased intake of fruit and vegetables, cardiovascular disease mortality was reduced by ~20% (84). By contrast, only the smallest two of seven vitamin C supplement studies included in a recent meta-analysis (85) have shown any benefit in reducing major cardiovascular events, leading to the conclusion that vitamin C has no effect on this end point. A large-scale study conducted over 20 years found that diets rich in vitamin C were associated with a lower incidence of stroke in elderly people (+65 years), but no significant association was found with coronary heart disease (86). Taken together, these findings infer that vitamin C might not be the active agent in the effect seen in the Norfolk trial, but that the benefits shown by eating sufficient fruit and vegetables to have a significant impact on plasma vitamin C induce a protective effect through other fruit and vegetable-derived nutrients. The Cochrane review on antioxidant supplements similarly concludes that vitamin C fails to reduce all-cause mortality (63).

### Vitamin E

Vitamin E is the most comprehensively studied antioxidant in humans, to date, particularly with respect to large-scale clinical trials. The results of these trials have had a major impact on the perception of antioxidants as whole in the cardiovascular arena.

#### Biochemistry

**Figure 3:**
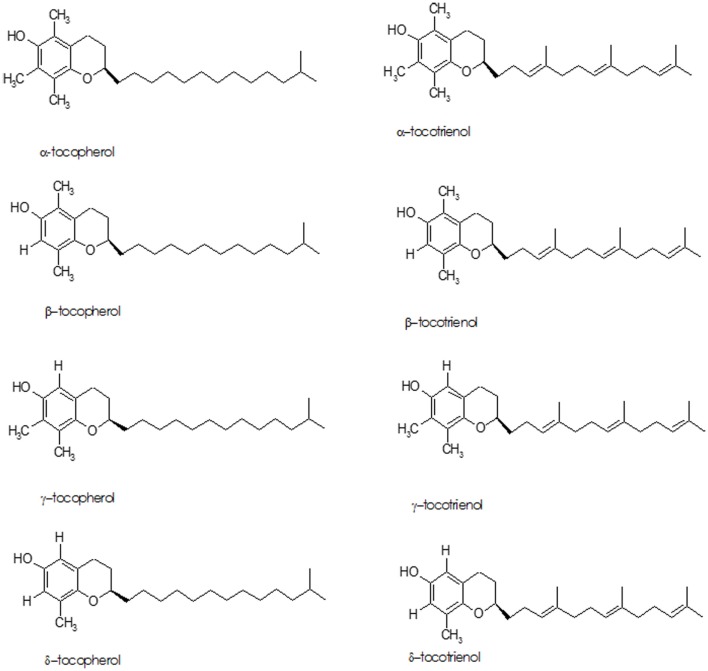
**Structure of tocopherols and tocotrienols**.

The term “Vitamin E” refers to eight lipid soluble, isomeric compounds containing three asymmetric carbon atoms, known as tocopherols and tocotrienols (Figure 3). The major isoforms in human diet are α- and γ-tocopherol; α-tocopherol is the best-researched as it is the most potent antioxidant of the group (87–89). Cooking oils, egg yolk, butter, green leafy vegetables, and some fruits (kiwi fruit, pumpkins, mangoes, papayas, and tomatoes) are rich sources of vitamin E. While vitamin E deficiency can cause serious health problems, it usually occurs in premature babies who have genetic deficiency in tocopherol transport protein or have fat absorption problems – it is rarely due to deficiency in the diet (90).

The tocopherols and tocotrienols are absorbed with fats through a non-specific uptake mechanism in the intestine, from where they are transported to the liver in chylomicrons prior to secretion in very low density lipoproteins (VLDL). VLDL is subject to delipidation and converted to LDL, enriching all lipoproteins with vitamin E. LDL transport is essential for delivering vitamin E into tissues; unbound vitamin E is hydrophobic, making it immiscible with the aqueous plasma and cellular compartments (87).

#### *In Vitro* and *In Vivo* Animal Studies

*In vitro* and *in vivo* animal experiments have led to the proposal that vitamin E, and α-tocopherol, in particular, has protective qualities that might help to prevent cardiovascular disease. *In vitro* studies have shown that α-tocopherol can protect against oxidative stress, inflammation, and endothelial dysfunction, all of which are characteristic of atherosclerotic plaque development. For example, monocyte adhesion to the endothelium can be reduced in the presence of α-tocopherol in cultured monocytes (91) and primary human monocytes (92). Vitamin E has also been shown to inhibit expression of adhesion molecules, VCAM-1 and ICAM-1, on cultured endothelial cells stimulated by ox-LDL (93). Vascular smooth muscle cell proliferation can also be inhibited by α-tocopherol via inhibition of protein kinase C activity (94).

The effects of vitamin E/α-tocopherol in *in vivo* animal models are inconclusive. For example, α-tocopherol can significantly lower circulating C reactive protein (CRP), an inflammatory marker that is associated with atherosclerosis (95) and, importantly from an antioxidant perspective, vitamin E supplementation can protect LDL from peroxidation, which may slow atherosclerotic plaque development (96). Lipid peroxidation can be prevented by α-tocopherol, through scavenging of ROS, preventing the early stages of atherosclerosis, and limiting the damage caused during ischemia reperfusion, where excessive ROS are produced (97, 98). In addition, vitamin E supplementation has been shown to reduce intravascular fatty deposits and macrophage activation in a mouse model of diabetes in which the animals are ordinarily prone to developing atherosclerosis (99). By contrast, however, vitamin E supplementation failed to reduce atherosclerotic lesion size or levels of 8-iso-prostaglandin F_2α_, a marker of oxidative stress, in obese hyperlipidaemic mice (100).

#### Clinical Studies and Trials

Vitamin E has been the subject of two large clinical trials: the Cambridge heart antioxidant study (CHAOS) and the heart outcomes prevention evaluation study (HOPE). The results from CHAOS showed some promise in that, while α-tocopherol supplementation failed to have an impact on cardiovascular mortality rates, it succeeded in reducing the incidence of non-fatal myocardial infarction in patients with coronary artery disease (101). Results from the HOPE trial, however, were disappointing, because vitamin E supplementation was found to have no effect on cardiovascular outcomes in patients at high risk of cardiovascular events or in patients with diabetes (102, 103). It is difficult to speculate on the reasons for this difference because both studies used patients with similar disease profiles, although the HOPE trial measured many more outcomes than CHAOS. The CHAOS trial only recruited patients with angiographically proven coronary artery atherosclerosis, whereas the HOPE trial recruited those at high risk of cardiovascular disease. Vitamin E was administered in the same way in both trials, but initially in CHAOS, a high dose (800 IU) was administered to the first group of patients recruited. Irrespective of the minor differences between individual studies, the antioxidant Cochrane review found that vitamin E supplements have the potential to significantly increase all-cause mortality (63).

Despite the disappointing outcomes of these large-scale trials, there is still a broad spectrum of *in vitro* and *in vivo* data that support the possibility that vitamin E may exert cardioprotective properties. For example, dietary supplementation with vitamin E has been shown to inhibit platelet adhesion *ex vivo*, suggesting a potential benefit in preventing thrombus formation (104). In any event, the results of the HOPE trial dealt a massive blow to vitamin E as a beneficial antioxidant in cardiovascular disease and led to a widespread sense that the whole concept of antioxidant therapy might be flawed. While the former conclusion might hold some truth, the latter perception is almost certainly unwarranted. The current recommendation from the US preventative task force is that vitamin E supplementation is not recommended in primary prevention of cardiovascular disease (62).

### Vitamin A and the carotenoids

#### Biochemistry

**Figure 4:**
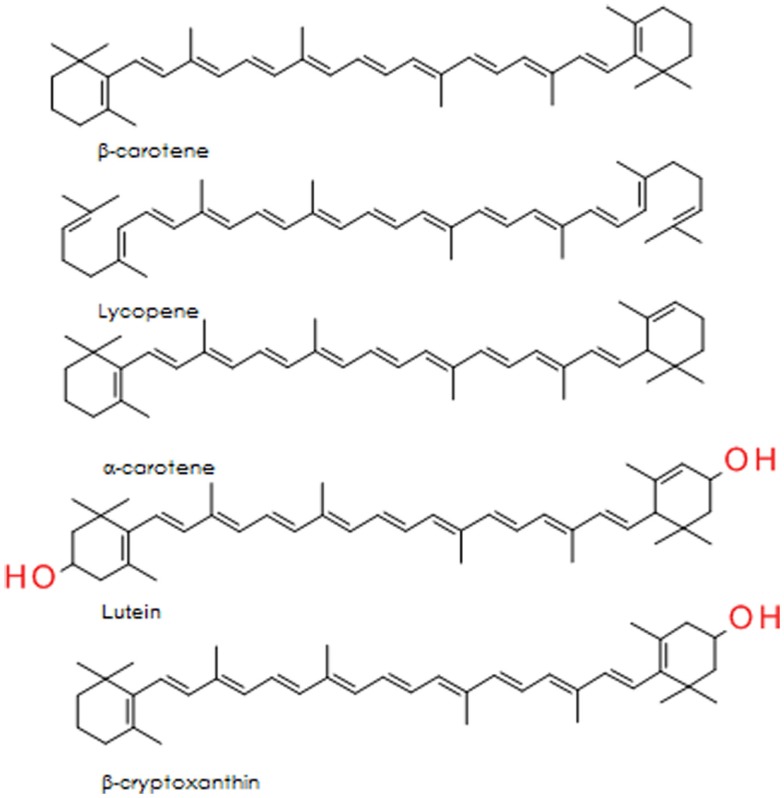
**Structure of five major carotenoids in human diet**.

The carotenoids are a group of lipid soluble, vibrantly colored pigments (yellow, orange, and red) found extensively in fruit and vegetables (Table 2). Retinol (vitamin A) can be synthesized from β-carotene in the gut, both before and after absorption [Ref. (105) for detailed review]. Lycopene, the precursor for β-carotene and many other carotenoids, is probably the most studied of this group. It is a potent singlet oxygen scavenger that is twice as effective in this capacity as β-carotene and 10 times more effective than α-tocopherol (106). The remarkable free radical scavenging activity of lycopene has been attributed to its highly unsaturated chemical identity, although it does not vary greatly from other carotenoids in this regard. The structures of the important carotenoids are illustrated in Figure 4.

**Table 2:** **Dietary sources of carotenoids**.

Carotenoid	Dietary source
β-carotene	Apricot, carrot, spinach, cantaloupe, broccoli, green beet, tomato
Lycopene	Tomato, guava, watermelon, pink grapefruit
α-carotene	Carrot
Lutein and zeaxanthin	Spinach, green beet, broccoli, green peas
β-crytoxanthin	Tangerine, papaya

While lycopene’s antioxidant activity might be higher than that of vitamin E and other diet-derived antioxidants, the limitation to *in vivo* antioxidant effects revolve around its bioavailability (plasma concentrations typically <1 μM in free living individuals in a placebo-controlled β-carotene trial) (107). However, the situation is more complex than simply considering raw plasma concentration because lycopene associates with LDL in the plasma, where the local concentration in these lipid-rich moieties will be considerably higher. In addition, the lipid solubility of lycopene ensures that it accumulates in fat-rich organs and tissues (e.g., liver, prostate, testes) (108), where it might reach sufficient concentrations to have a genuine impact on antioxidant capacity. As with other diet-derived components, it is also crucial to consider the effect of cooking and oxidation prior to ingestion, as well as metabolism on the eventual metabolites available in the appropriate compartment to influence biological processes. The impact of cooking is not necessarily detrimental to antioxidant capacity, studies involving thermal processing of tomatoes indicated that heating reduced vitamin C content, but caused a concomitant increase in bioavailable lycopene, possibly due to release from the matrix of the fruit, and a net increase in antioxidant capacity (109).

#### *In Vitro* Studies

Evidence surrounding carotenoids from *in vitro* studies is conflicting. For example, β-carotene, lycopene, and lutein all prevent Cu^2+^-mediated LDL peroxidation (110). However, a similar study using endothelial cells to induce lipid peroxidation found no benefit from β-carotene or lycopene enrichment of LDL (111). Both of these studies used similar concentrations of carotenoids; therefore, it is most likely that different results stem from using different models to induce oxidative stress.

*In vitro* studies indicate that β-carotene and lycopene are capable of reducing plasma cholesterol levels through inhibition of HMG-CoA reductase, the rate-limiting enzyme involved in cholesterol synthesis. HMG-CoA reductase is the target of the highly successful group of drugs known as statins (e.g., simvastatin, fluvastatin) and therefore constitutes a valid target for plant-derived agents. Both β-carotene and lycopene (10 μM) reduced HMG-CoA activity to a similar extent as fluvastatin (10 μg/ml), suggesting a potential therapeutic application for carotenoids. In addition to this, macrophage LDL receptor activity was increased in the presence of these carotenoids, reducing circulating LDL, suggesting a possible a protective role in the cardiovascular system (112, 113). By contrast, however, it has been demonstrated in cardiac myocytes and endothelial cells that retinoic acid (the active metabolite of retinol) has been shown to reduce activity of inducible NOS (iNOS), which is usually active during inflammation, producing cytotoxic levels of NO in both cardiac myocytes and endothelial cells (114).

#### Clinical Studies and Trials

Many clinical studies have suggested that carotenoids have cardioprotective properties. The Physicians Health Study found that coronary artery disease was less prevalent in men who ate vegetables rich in carotenoids (115). Moreover, recent studies, such as the coronary artery risk development in young adults (CARDIA) and the young adult longitudinal trends in antioxidants (YALTA) studies, have found that high-plasma carotenoid concentrations are associated with reduced inflammation, oxidative stress, and endothelial dysfunction, three important characteristics of atherosclerosis (116), while the Bruneck study found a lower incidence of atherosclerosis in individuals with higher plasma levels of β-carotene and α-carotene (117). Reduction in markers of endothelial dysfunction, such as soluble intercellular adhesion molecule (sICAM-1), and inflammation (CRP) are associated with elevated plasma concentrations of carotenoids resulting from high intake of fruit and vegetables rich in carotenoids (118). By contrast, however, the β-carotene and retinol efficacy trial (CARET) found an inverse correlation between supplementation and risk of cardiovascular disease; this is the only trial, to date, which has investigated the impact of retinol supplementation on cardiovascular disease, albeit in combination with β-carotene (119). The EURAMIC study found a weak association between adipose β-carotene concentration and risk of MI, but the effect was lost after controlling for a range of confounding factors (120). Findings from a more recent study in 1031 Finnish men, however, found a more convincing correlation between serum β-carotene and reduction in risk of MI (121). Similarly, LDL oxidation was not reduced by β-carotene in a study in which vitamin E was found to be beneficial (96). Again, there is perhaps a disparity between findings where β-carotene is used as a marker of fruit and vegetable ingestion and those involving supplements, which could be interpreted to indicate that β-carotene is a bystander rather than causal in dietary studies. Indeed, based on these studies, the statement from the US Preventative Task Force is to not recommend β-carotene for prevention of cardiovascular disease (62). Likewise, the Cochrane review on antioxidant supplements and all-cause mortality found β-carotene and vitamin A to significantly increase all-cause mortality (63).

There has been considerable recent interest in lycopene – the carotenoid that is found in high concentration in tomatoes. In the EURAMIC study, adipose lycopene concentrations correlated with a reduction in risk of MI and the effect was retained after correction for confounding factors. The odds ratio was modeled in this study using the contrast between the 10th and 90th percentile for adipose lycopene and was found to be 0.52 (120), suggesting that risk of mortality was substantially reduced in those with high-adipose lycopene. A similar correlation has been reported for serum lycopene and a reduction in risk of MI (121). These promising epidemiological studies have prompted a number of small interventional studies, which have generally shown beneficial effects of lycopene on a range of outcome measures related to cardiovascular disease [see Ref. (122) for review]. In short, the data indicate that diet- or supplement-derived lycopene reduces inflammation and oxidative stress markers in overweight and healthy individuals, improves endothelial function, and reduces platelet activity (123, 124). Interestingly, the most recent study (125) found that lycopene only improved endothelial function in a patient group and not a parallel healthy volunteer group, perhaps suggesting a therapeutic rather than preventative role for lycopene. All of these studies are small and require confirmation in larger cohorts, but the findings are positive and the anti-thrombotic data (123, 124) were the basis for a patent and the subsequent commercialization of a tomato extract with European Food Standard Agency approval for therapeutic claims.

Compared to β-carotene and lycopene, the other carotenoids have received considerably less attention. The Los Angeles Atherosclerosis Study demonstrated that lutein-rich diets were inversely related to early atherosclerotic lesion development. Moreover, *in vitro* and knockout mice findings were also presented showing lutein reduced monocyte chemotaxis in an *in vitro* atherosclerotic model and atherosclerotic lesion size was reduced in lutein-treated apolipoprotein E (Apo-E) and LDL receptor knockout mice (126). Zeaxanthin and β-cryptoxanthin have not been as intensely investigated as β-carotene, lycopene, and lutein (127), although plasma lutein, zeaxanthin, and β-cryptoxanthin (collectively known as the oxygenated carotenoids) have been shown to be inversely associated with coronary artery disease (128).

Despite encouraging *in vitro* and *in vivo* data relating to a range of carotenoids, and an apparent correlation between plasma carotenoid concentration and reduced risk of cardiovascular disease in epidemiological studies, data from carotenoid intervention trials have failed to provide incontrovertible evidence in support of carotenoids in cardiovascular disease. However, the findings have not all been negative and the results relating to lycopene in particular suggest that the less convincing findings for β-carotene should not be taken as indicative of all carotenoids. Indeed, the *ex vivo* and *in vitro* data for tomato extracts have been regarded to be sufficiently strong by the European regulatory body to gain rare approval for therapeutic claims.

### Folate and other B vitamins

#### Biochemistry

**Figure 5:**
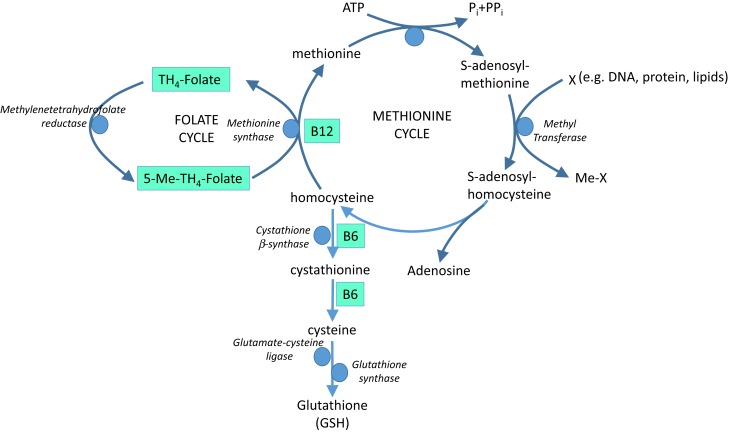
**The role of vitamins B9 (folate), B6, and B12 in methionine metabolism and glutathione (GSH) synthesis**.

Dark leafy vegetables, such as spinach, are rich sources of folate (folic acid, vitamin B9) and other B vitamins. Folate and the B vitamins have a crucial role in metabolism of the essential amino acid, methionine, with impacts on both homocysteine and antioxidant GSH (Figure 5).

#### *In Vitro* and *In Vivo* Animal Studies

Homocysteine is believed to induce endothelial dysfunction because its thiol group is easily oxidized, generating ROS, which reduce bioavailability of NO by direct oxidative inactivation and depletion of intracellular GSH pools (129, 130). In addition, homocysteine has been implicated in increased formation of asymmetric dimethyl-l-arginine, an endogenous inhibitor of NOS (131) and in protein kinase C activation, leading to the inhibition of eNOS and further reducing NO bioavailability (132). Furthermore, vascular inflammation can be promoted by homocysteine through NF-κB-mediated expression of the pro-inflammatory cytokines, monocyte chemotactic protein (MCP-1), and IL-8 (129). Hyperhomocysteinemia is therefore recognized to be an independent risk factor for cardiovascular disease (133), at least partly via oxidative stress. Homocysteine-lowering therapy is a potential option in homocysteine-driven cardiovascular dysfunction; B vitamins involved in homocysteine cycling and GSH synthesis (Figure 5) represent potential therapeutic agents in this arena.

Folate has been demonstrated to scavenge hydroxyl and lipid peroxyl radicals *in vitro* (134). The latter is perhaps surprising, given the water solubility of folic acid but, if true, is likely to contribute to any protective effects in cardiovascular disease. Moreover, folate supplementation *in vivo* in Apo-E knockout mice (135) has been shown to decrease number of atherosclerotic plaques in these animals, and was also associated with a decrease in LDL peroxidation. Similar results have been reported in a homocysteine-induced rat model of atherosclerosis (136), while a further potential use of folate has been identified in an animal model of abdominal aortic aneurysm (137), whereby folate acts to help reverse uncoupling of eNOS implicated in the etiology of the condition.

#### Clinical Studies and Trials

Low-circulating levels of folate, vitamin B12, and vitamin B6 are risk factors for stroke, peripheral vascular disease, and coronary artery disease (138). Supplementation of folate, hydroxocobalamin (vitamin B12), and pyridoxine (vitamin B6) reduces serum homocysteine in elderly patients with slightly elevated plasma concentrations (139). A similar study found that folate, hydroxocobalamin, and pyridoxine supplements for 8 weeks reduced serum homocysteine to within the normal range in healthy volunteers and in patients with venous thrombosis (140). Moreover, a similar small-scale supplementation study found a decrease in carotid artery intima-media thickness in high-risk cerebral infarction patients (141). A further positive study showed that low-dose folate supplementation improved endothelial function in patients with cardiovascular disease shortly after receiving a coronary graft. Importantly, the benefits in this study were attributed to improved coupling of endothelial NO synthase through the essential cofactor, tetrahydrobiopterin, rather than by an impact on plasma homocysteine levels (142).

The vitamin intervention for stroke prevention (VISP) randomized controlled trial found no significant effect of folate, hydroxocobalamin, and pyridoxine supplementation in reducing incidence of cerebral infarction, coronary events, or cardiovascular death. Patients with non-disabling cerebral infarction received a high-dose or low-dose folate, hydroxocobalamin, and pyridoxine supplementation for 2 years, after which recurrent cerebral infarction, coronary events, or death was recorded (143).

As with many of the other antioxidant vitamin interventions discussed above, there appears to be a mis-match between the potential of vitamin B supplements shown *in vitro* and suggested by cohort, prospective, and retrospective clinical studies, and randomized control trials with B vitamins. In a recent review on the subject (144), a convincing argument is put forward that any beneficial effects of vitamin B (or vitamin E) in intervention studies is masked by the effects of statins, aspirin, and other drugs that patient groups are inevitably receiving. Added to this, we would argue that vitamin therapy is only likely to benefit individuals deficient in the vitamin of choice and could only show benefit in a sub-set of the study populations. The findings of a Cochrane systematic review for vitamin B6, 9, and 12 in cardiovascular disease were that there is no evidence to support the use of these B vitamins to prevent cardiovascular events (145).

### Polyphenolic compounds

#### Biochemistry

**Figure 6:**
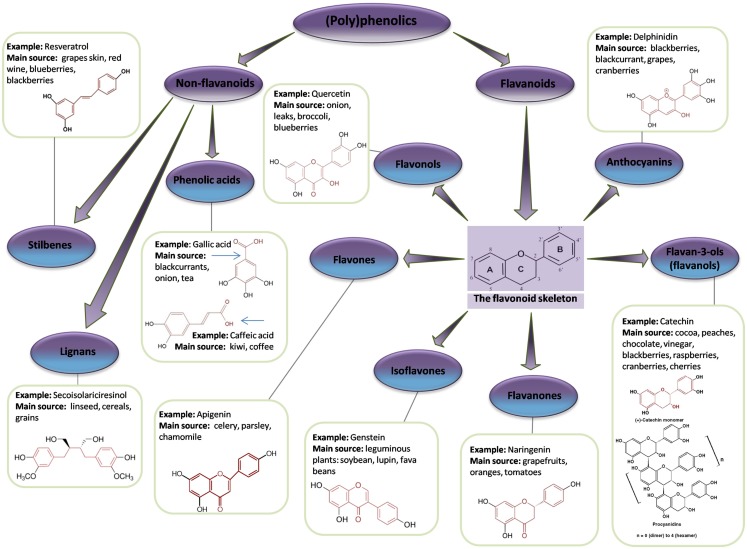
**Flavonoid and non-flavonoid phenolic compounds**. Schematic showing phenolic compounds, along with typical examples and associated chemical structure (adapted from http://www.frenchglory.com/polyphenol-classification.html).

A polyphenol is defined as a compound that contains two or more phenol groups (146). This large group of compounds is divided into several sub-groups: flavonoids, phenolic acids, stilbenes, and lignans (147) (Figure 6). Polyphenolic compounds are abundant in plants and are readily found in fruit and vegetables (Table 3). In addition, they are important components of herbs and spices and are likely to be critical ingredients in Chinese medicines.

**Table 3:** **Dietary sources of common (poly)phenolic compounds [adapted from Watson et al. (148)]**.

Polyphenol class	Examples	Common dietary sources
Anthocyanidins	Cyanidin, delphinidin, malvidin, pelargonidin, peonidin, petunidin	Fruit, red wine, some cereals, aubergines, cabbage, beans, onions, radishes
Catechins	Catechin, epicatechin, gallocatechin, epigallocatechin	Tea (black and green), cocoa, chocolate
Flavanones	Hesperitin, hesperidin, homoeriodictyol, naringenin, naringin	Citrus fruit, tomatoes, mint
Flavones	Apigenin, luteolin, tangeritin, nobiletin, sinensetin	Fruit, vegetables, some cereals, skin of citrus fruit
Flavonols	Kaempferol, myricetin, quercitin	Fruit, onions, kale, leeks, broccoli, blueberries, red wine, tea
Isoflavones	Daidzein, genistein, glycitein	Soybeans, peanuts, leguminous plants
Hydroxybenzoic acids	Gallic acid, *p*-hydroxybenzoic acid, vanillic acid	Tea, wheat, raspberry, blackcurrant, strawberry
Hydroxycinnamic acids	Caffeic acid, ferulic acid, *p*-coumaric acid, sinapic acid	Kiwifruit, blueberries, apples, cereals
Lignans	Pinoresinol, podophyllotoxin, steganacin	Flax seed, sesame seeds, some cereals, broccoli, cabbage, apricots, strawberries
Stilbenes	Resveratrol	Red wine
Tannins	Castalin, pentagalloyl glucose, procyanidins	Tea, berries, wine, cocoa, chocolate

##### Polyphenols as antioxidants

The paradigm for the mechanism of action of polyphenols is through direct antioxidant activity, on account of their interaction with ROS, including both radical and non-radical oxygen species, such as ${\text{O}}_{2}^{\cdot \;-}$, H_2_O_2_, HOCl, NO, as well as those derived from biomolecules, such as LDL, proteins, and oligonucleic acids (DNA, RNA) (149–152). Structural features, such as number and relative position of hydroxyl and catechol groups, determine the antioxidant properties of polyphenols (150, 152, 153).

Polyphenols can also act as antioxidants by chelating metal ions, such as iron and copper, which are involved in conversion of ${\text{O}}_{2}^{\cdot \;-}$ and H_2_O_2_ into highly aggressive ^⋅^OH (Fenton chemistry). They can also block the action of some enzymes responsible for generation of superoxide radical, such as xanthine oxidase (XO) and protein kinase C, as well as activating antioxidant enzymes (150, 154).

There is an ongoing debate regarding the probability of observed direct antioxidant activities *in vivo*, since supra-physiological concentrations (100–400 μM) of polyphenols were often used in tests determining their antioxidant properties. Low bioavailability (~1 μM) of phenolic compounds seriously questions their ability to engage in direct antioxidant potential in the *in vivo* scenario (152). The emerging concept is that phenolics may induce endogenous antioxidant defense systems through modulation of gene expression, therefore, protecting against oxidative stress in an indirect manner (154, 155).

##### Polyphenols as pro-oxidants

The pro-oxidant activities of polyphenols are based on their ability to generate ROS. It has been reported that phenolic compounds can oxidize readily in beverages, tissue culture media, and phosphate buffers, especially in the presence of transition metal ions (156, 157). Oxidation of polyphenols leads to superoxide radical and H_2_O_2_ production, as well as a complex mixture of semiquinones and quinones, all of which are potentially toxic (157, 158). The level of cytotoxicity depends on both the type of polyphenol and the amount of free radicals generated (156).

A variety of polyphenols and polyphenol-containing extracts have been reported to generate ROS, including green tea, black tea, and apple extracts, as well as individual phenolics, such as gallic acid, protocatechuic acid, vanillic acid, ellagic acid, caffeic acid, quercetin, rutin, kaempferol, catechin, epicatechin, delphinidin, and malvidin (158).

The pro-oxidant effects of polyphenols can also be beneficial, since, by imposing a mild degree of oxidative stress, they can induce endogenous antioxidant defense mechanisms. However, the amount of research on the antioxidant potential of polyphenols overshadows the lesser number of studies on the biological implications of their pro-oxidant properties (154, 156).

Fruit and vegetable-derived polyphenols have been subject to a great deal of study, providing a substantial body of evidence to support the beneficial effects of these substances on health. Here, we look specifically at the benefits of polyphenols on the cardiovascular system (159), where polyphenols have been found to be associated with a reduction in mortality from cardiovascular disease (160–162), but the mechanisms through which they exert their cardioprotective actions are not yet fully understood (163). The assumption is that the antioxidant properties of polyphenols protect blood vessels against oxidative damage and the associated cardiovascular risk factors (163, 164). It has been proposed that polyphenols might protect vascular endothelial cells against ROS-induced damage, thereby preventing oxidation of LDL and protecting the crucial vasodilator nitric oxide (NO) against oxidation (164–167).

The objection to the direct antioxidant paradigm to explain the beneficial effects of polyphenols in vascular diseases is that polyphenols are characterized by low bioavailability; the concentrations required to induce direct antioxidant activity are infeasible *in vivo*. Instead, it is becoming increasingly recognized that phenolic compounds can interact with various molecular targets and affect multiple signaling pathways in endothelial cells, providing an alternative putative mechanism to define the means by which these agents can induce benefit despite very low concentrations *in vivo* (150). Prominent among the putative mechanisms triggered by low concentrations of polyphenols is activation of the nuclear factor E2-related factor 2 (Nrf2), antioxidant response element (ARE) pathway (168). Activation of Nrf2 causes it to bind to the enhancer DNA sequence, ARE, which regulates more than 200 genes that code for a wide variety of proteins, including antioxidant and detoxifying enzymes. The key antioxidant enzymes induced are glutamyl-cysteine ligase (GCL – the rate-limiting enzyme for GSH synthesis), GPx, thioredoxin, thioredoxin reductase, SOD, and heme oxygenase-1 (HO-1). A wide variety of polyphenols have been identified to be effective activators of this pathway, with the effect of driving a broad spectrum antioxidant response [for review, see Ref. (169)]. In addition, phenolic compounds impact on the cardiovascular system by improving endothelial function, inhibiting angiogenesis, cell migration, and proliferation of blood vessels, and reducing platelet aggregation and hypertension (164, 166, 170). Plant polyphenols may stimulate the production of major vasodilatory factors [NO, endothelium-derived hyperpolarizing factor (EDHF) and prostacyclin] and inhibit the synthesis of vasoconstrictor endothelin-1 in endothelial cells (171). Moreover, phenolic compounds inhibit the expression of two major pro-angiogenic factors, vascular endothelial growth factor (VEGF), and matrix metalloproteinase-2 (MMP-2) in smooth muscle cells (172).

In endothelial cells, one of the mechanisms that drives the effects described above involves Ca^2+^-mediated activation of the phosphoinositide 3 (PI3)-kinase/Akt pathway, leading to rapid enhanced expression and stimulation of eNOS, as well as formation of EDHF. In smooth muscle cells, polyphenols have been shown to induce both redox-sensitive inhibition of the p38 mitogen-activated protein kinase (p38 MAPK) pathway activation, which leads to inhibition of platelet-derived growth factor (PDGF)-induced VEGF gene expression, and other redox-insensitive mechanisms (171, 172). While these effects have been seen with a range of polyphenols, the cellular response and its intensity may vary between phenolic compounds (142, 158). In addition, it is important to note that the concentrations used in many of these experiments are in the micromolar range.

##### Bioavailability of (Poly)phenolics

Polyphenols are characterized by very low bioavailability, with <1% of the ingested amount reaching the plasma (162, 173–176); concentrations in human plasma usually peak at 0.5–2 h after ingestion, falling to near baseline levels within 8–12 h (177). Maximum concentrations of polyphenols found in the blood after consumption of polyphenol-rich foods or beverages tend to be around 0.1–1 μM. This is in stark contrast to the concentrations regularly used *in vitro*, where supra-physiological concentrations of individual polyphenols or extracts tend to be in the micromolar range (151, 160, 178).

Numerous polyphenols have been detected in the plasma at concentrations around 1 μM in their native intact forms (glucosides), as an effect of fast absorption in the stomach; these are rapidly excreted (179). Anthocyanin glycosides pass through the stomach and are hydrolyzed in the small intestine or by colon microbiota (180). Liberated aglycones are very unstable and undergo further degradation to simple phenolic acids and aldehydes at neutral pH. Smaller phenolic species and other metabolites have greater chemical stability in the physiological environment than anthocyanins, suggesting that the metabolites and not the parent compounds might be present in the plasma at much higher concentrations and could, therefore, play an important role in physiological effects (162). There needs now to be a concerted effort to identify the *in vivo* metabolite profile derived from given polyphenols to guide more appropriate *in vitro* experiments that mimic metabolite composition and concentration. This is a major limitation of *in vitro* experiments that utilize crude extracts.

#### *In Vitro* and *In Vivo* Animal Studies

There are three major mechanisms by which (poly)phenols might impact on cardiovascular disease, acting as antioxidants, anti-inflammatory agents, and/or anti-thrombotic agents.

The high levels of polyphenolics contribute to the high-antioxidant capacity in fruit and vegetables (181, 182) and extracts from plants or fruits with high-antioxidant capacity have powerful free radical scavenging activity *in vitro*. Apples are a rich source of polyphenols, including chlorogenic acid, epicatechin, procyanidin B_2_, phloretin, and quercetin. The antioxidant capacity, determined by a ferric reducing ability of plasma (FRAP) assay, has been shown to vary between apple genotype, and has been demonstrated to relate directly to the polyphenol content of the fruit (182). Similarly, currants, rich in vanillic acid, have shown a similar pattern, using the 1,1-diphenyl-2-picrylhydrazyl (DPPH^.^) radical scavenging assay (183). Meanwhile, the flavonols, quercetin and rutin, are potent scavengers of peroxynitrite, the highly noxious RNS formed by the reaction between superoxide and NO (184). Peroxynitrite has been shown to convert LDL to ox-LDL, key to the development of atherosclerosis, and to oxidize α-tocopherol (185). Few, if any of these *in vitro* experiments use concentrations that equate to those likely to be bioavailable through oral ingestion; so, these findings should be viewed with caution.

The anti-atherogenic effects of the polyphenolics include preventing lipid peroxidation (186) and uptake of ox-LDL by macrophages (187), increasing NO bioavailability (188, 189), and decreasing activation of redox-sensitive genes (190). Polyphenolic compounds present in pomegranate juice, red wine, and grapes prevent ox-LDL deposition in the vascular wall. Purple grape juice can both decrease LDL oxidation and flow-mediated vasodilation in patients with coronary artery disease (191). Resveratrol, cinnamic and hydroxycinnamic acids, and cyanidin present in these sources can increase expression of eNOS (188, 189, 192).

Resveratrol, a stilbene found in red or purple grapes, has also been shown to have potent anti-inflammatory properties. Vascular cell adhesion molecule-1 (VCAM-1), E-selectin, and intracellular adhesion molecule-1 (ICAM-1) are all markers of inflammation that are down-regulated endothelial cells, cultured in the presence of non-physiological concentrations of resveratrol (1–100 μM) (193). Moreover, resveratrol has been shown to inhibit lipopolysaccharide (LPS)-induced monocyte adhesion to cultured endothelial cells (193). However, many of the positive aspects of the results obtained so far with resveratrol are tempered by the fact that the plasma concentration of resveratrol is thought to be very low (picomolar to nanomolar), mainly because of its rapid metabolism in the gut. To reach the concentrations used in many experiments, 25–50 mg of resveratrol would need to be consumed, the equivalent of drinking 20 glasses of red wine a day (194, 195). It is somewhat difficult to explain, therefore, how the acute effects of cigarette smoking on endothelial dysfunction can be reversed by direct antioxidant activity attributed to a single glass of red wine, irrespective of alcohol content (196). Given the above information, it is unlikely that the results are due solely to resveratrol, but they might nevertheless be due to the combined effects of antioxidants in red wine. The same paradigm might also apply to fruit and vegetable consumption; perhaps a single component is not wholly responsible for the beneficial effects seen. Alternatively, the antioxidant benefits of resveratrol *in vivo* might be entirely driven by stimulation of antioxidant signaling pathways (e.g., activation of the Nrf2/ARE pathway), requiring substantially lower concentrations to be effective.

Delphinidin, an anthocyanin found in berries, has anti-apoptotic properties in cultured endothelial cells; it is thought that NO may be involved, as this effect is prevented in the presence of NOS inhibitors, leading to the suggestion that delphinidin may exhibit cardioprotection by preventing endothelial cell apoptosis (197). The anthocyanins have strong anti-thrombotic activities (198, 199): anthocyanins found in fruit and vegetables can inhibit P-selectin expression, an adhesion molecule involved in platelet activation. Resting, TRAP-activated, H_2_O_2_-stressed, and adrenaline pre-activated platelets were inhibited by the anthocyanins *in vitro*, whereas collagen- and ADP-activation are not affected (198). It is plausible that anthocyanins in purple grape juice are at least partly responsible for its ability to inhibit platelet function both *in vitro* and *in vivo*, through an increase in NO production and a decrease in superoxide generation. Analysis of purple grape juice using high-performance liquid chromatography (HPLC) found that fractions containing high amounts of pro-anthocyanins were most effective in preventing lipid peroxidation (199, 200).

Quercetin has anti-hypertensive properties and reduces cardiac hypertrophy in animal models. Hypertension has been shown to be reversed in spontaneously hypertensive rats and Goldblatt hypertensive rats, but not in normotensive controls (201, 202). In addition, cardiac hypertrophy in hypertensive rats was reduced in animals fed a quercetin-rich diet (203, 204). A wide range of studies have indicated that quercetin has the capability to act as an antioxidant *in vivo* through altered gene transcription (205–208). These *in vivo* experiments are likely to be a more reliable source of mechanistic information than *in vitro* experiments involving crude extracts and isolated compounds, often at inappropriate concentrations.

#### Clinical Studies and Trials

Epidemiological studies appear to show an association between high-flavonoid intake and cardiovascular outcomes. For example, the Zupthen Elderly study found a significant inverse relationship between flavonoid intake and coronary heart disease after 5 years, but after 10 years this relationship was no longer significant (209, 210). Moreover, the Rotterdam study found a significant inverse relationship between total flavonoid intake from the diet, in particular, black tea (which is rich in flavon-3-ols in particular), with myocardial infarction incidence (211).

A meta-analysis published in 2008 reported on 133 placebo-controlled trials on specific flavonoids and flavonoid-rich foods (212). None of the studies reported on morbidity or mortality; instead, they measured one or more of a range of physiological measures (blood pressure, endothelial function) and markers (e.g., LDL) of cardiovascular disease. The heterogeneity of the studies made firm conclusions difficult to reach, but certain trends were identified in this meta-analysis. Measures of flow-mediated dilatation – an indicator of endothelial function – were universally improved in the acute phase, irrespective of the epicatechin dose administered, but there was little effect of flavonoid interventions on either blood pressure or LDL cholesterol (212).

More recent trials on specific polyphenols or food types have lent weight to the concept that there might be merit in polyphenol supplementation in cardiovascular disease prevention. In a modest placebo-controlled crossover clinical trial involving 93 obese or overweight men with metabolic syndrome traits, quercetin was found to reduce systolic blood pressure by ~3 mmHg, an effect that was more pronounced in the sub-group of patients with hypertension (213). Despite the low-plasma concentrations of quercetin measured in this study (71–269 nM) and a lack of significant increase in plasma antioxidant capacity, there was also a significant increase in HDL and a reduction in the concentration of pro-atherogenic ox-LDL.

However, the studies that have captured the imagination of the press and public the most are those involving the benefits of tea, chocolate, and red wine; it is evidently attractive for healthy nutritional interventions to also be pleasurable. The phenolic content of chocolate and both green and black tea is dominated by flavan-3-ols (catechin and epicatechin) and isoflavones. Dietary intake of cocoa or chocolate is inversely associated with carotid atherosclerosis (214) and calcified plaque development (215). In a double-blind placebo-controlled trial with flavonoid-enriched chocolate in patients with type 2 diabetes, there was an improvement in carotid vascular function (216). Similarly, isoflavone-specific intervention trials in healthy individuals have indicated some benefits with respect to endothelial function, blood pressure, and arterial stiffness. However, the longest trial with isoflavones (2.7-year follow up) showed no benefit, as measured by common carotid artery intima-medial thickness.

Red wine is a flavonoid-rich drink that has long been associated with cardiovascular benefit. Its notoriety in this regard was triggered by the suggestion that high red wine consumption in France provided a plausible explanation for the so-called French paradox – the remarkably low risk of cardiovascular mortality among the French population, irrespective of high-saturated fat consumption and smoking rates. However simplistic the original suggestion was, the potential merits of red wine have been supported by data from the Copenhagen City Heart Study, in which individuals who drank moderate amounts (three to five glasses per day) of red wine were at substantially lower risk (RR – 0.51) of cardiovascular mortality than those who never drank wine (217). By contrast, there was no significant benefit from drinking beer and a substantial increase in risk associated with drinking spirits. Several recent intervention studies have interrogated the association with a view to establishing mechanism; the findings generally confirm beneficial effects, most likely conveyed by polyphenol constituents [but not necessarily resveratrol (218)]. However, the data regarding impact of red wine on antioxidant capacity (219) or platelet activity (220) are unconvincing, compared to benefits mediated via improvements in plasma lipoprotein profile (221, 222), NO concentration (223), and inflammatory markers [most notably chemokine C–C motif ligand 5 (CCL-5) (224) and NF-κB (219)]. Interestingly, there is a pattern emerging to suggest that alcohol-free red wine might convey enhanced benefits over red wine itself (222, 223).

Although the results from intervention studies are not universally supportive of benefit of polyphenol-rich foods and drinks, there is reason for optimism regarding potential benefits with respect to several markers of key elements (e.g., endothelial function, platelet function) central to the atherothrombotic process. Antioxidant activity is not necessarily a factor. Data relating to red wine consumption are perhaps the most convincing, with an association identified between moderate red wine consumption and cardiovascular mortality. However, it is also clear that the limited bioavailability of beneficial flavonoids is universally low (typically in the nanomolar range) and that a direct antioxidant effect is unlikely to drive the benefits seen. This does not, however, preclude the possibility that the beneficial effects are mediated by an adaptive response that includes up-regulation of endogenous antioxidant systems. In addition, it is apparent that flavonoids have subtle, antioxidant-independent effects (e.g., reduced inflammation, reduced insulin resistance), which might be at least as important as any antioxidant activity. It is also important to note that flavonoids are typically bitter to taste and that the strategy often employed in nature (berries) and in food production (chocolate) is to modulate the bitter taste with sugar (berries, chocolate) or alcohol (red wine). Inevitably, therefore, there is an element of risk balance between the benefits of flavonoids and the potential detrimental effects of associated ingredients in tasty foods and drinks.

### Minerals

A number of minerals have the potential for antioxidant activity *in vivo*, not through direct scavenging of free radicals (indeed, many transition metal ions are considered to be pro-oxidant), but instead through their requirement for antioxidant enzyme function. The requirement for metal ions, such as copper, zinc (cofactors for cytoplasmic SOD-1), manganese (cofactor for mitochondrial SOD-2), and selenium (cofactor for GPx and thioredoxins), is generally at trace levels that are easily obtainable through the diet. Deficiencies are, therefore, rare, but can occur in cases of malabsorption or in geographical regions where these minerals are deficient or absent in the soil. Of the minerals named above, selenium is the most recognized to be deficient in some populations, and to have a link to cardiovascular (and other) diseases (225), potentially on account of reduced antioxidant capacity.

#### Selenium

Selenium is required at trace levels and excessive supplementation can lead to toxicity (selenosis). Much of the work relating to selenium has centered on its role in cancer prevention, but a number of studies have focused on cardiovascular disease or have included cardiovascular outcomes as a sub-set of all-cause mortality. While observational studies suggest an inverse relationship between selenium levels in the body and cardiovascular risk (226), placebo-controlled large trials failed to find a significant effect of selenium supplements for CVD events (fatal and non-fatal; RR 1.03) (227), suggesting that selenium supplementation is ineffective in this setting. Indeed, the findings of the Cochrane review on antioxidants and all-cause mortality indicate that there is no evidence for reduced mortality with selenium supplementation. In this sense, the story for selenium mirrors those for many of the antioxidants above in that the results of observational studies are not supported by those from placebo-controlled trials. Once again, the lack of agreement between the two approaches might simply reflect the heterogeneity of the controlled trial population with respect to selenium levels; only a small sub-set might be deficient and likely to benefit from the intervention.

Keshan disease is a specific endemic cardiomyopathy has also been linked with selenium deficiency. The name relates to the region of China associated with an unusually high incidence of the disease, first identified in 1935. However, cases are found in a range of areas, predominantly in China, where the soil is deficient in selenium. The symptoms include acute or chronic episodes of cardiogenic shock and/or heart failure. Although the cause has not yet been fully characterized, animal studies suggest a possible mechanism involving reduced expression of the antioxidant enzyme, GPx1, leading to increased virulence of viruses associated with myocarditis (228). Clinical studies suggest an extra layer of complexity surrounding Keshan disease on account of decreased GPx1 activity in individuals with both selenium deficiency and a specific leucine-containing GPx1 allele (229). The link to selenium deficiency has long been recognized (230) and selenium supplementation trials have shown a striking impact on Keshan disease development and disease-related deaths, possibly on account of increased GPx activity (231). Here, at least, there is clear evidence of a substantial beneficial effect of a supplement that has the potential to increase antioxidant defense via GPx enzymes.

### Repurposed drugs as antioxidants

#### *N*-Acetylcysteine

*N*-Acetylcysteine (NAC) has sparked interest in the cardiovascular field on account of its reputation as an antioxidant. NAC is a drug that is licensed for use in several clinical settings, but it is also feely available as an oral supplement through health food outlets, although it is not generally considered to be a dietary component. In reality, its antioxidant activity and oral bioavailability is too low to merit consideration as an antioxidant *in vivo* [reviewed in Ref. (232)], but it nevertheless merits inclusion in this review on account of its ability to serve as a substrate for the synthesis of the critical ubiquitous intracellular antioxidant, GSH (233). GSH is central to a variety of important processes, including detoxification, and its synthesis, utilization, and redox status is under the regulation of a battery of enzymes, underlining its importance to cell function. GSH can be depleted in cells for a variety of reasons, most notably in hepatocytes as a result of paracetamol overdose, and also in association with atherosclerosis (41). NAC was originally designed as a means of effectively delivering substrate (cysteine) to the rate-limiting enzyme (glutamyl-cysteine ligase) for GSH synthesis. Although the mechanism by which NAC achieves this goal is not yet fully understood, it is certainly an effective remedy in patients suffering from paracetamol overdose (234). NAC is also used in some pulmonary conditions, but from an antioxidant perspective, it has received attention as a potential means of protecting against oxidative stress-induced radiocontrast nephropathy and as a possible alternative anti-thrombotic agent in diabetes (232). The latter use is the most relevant to this review and is supported by several *in vitro* and *in vivo* studies, suggesting that there is some merit in this concept. NAC has been shown to have a significant impact on blood pressure in patients with type 2 diabetes (235) and, most recently, a small clinical study has indicated that oral NAC supplementation in patients with type 2 diabetes reduces platelet–monocyte interaction (236), which is recognized to be a marker and potential mediator of cardiovascular disease. Importantly, the beneficial effect was most prominent in those deficient in GSH at baseline, indicating that, like vitamin C, cellular GSH is under strict regulation and providing more substrate for its synthesis to replenish cells is ineffectual. That said, GSH is well known to be depleted in diabetes (237), and *in vitro* studies indicate that NAC can replenish platelet GSH (238). Therefore, NAC might well present an effective strategy in this high-risk patient group, especially given its beneficial effect in blood vessels as well as platelets. Larger trials are merited to test whether these findings translate into improved cardiovascular outcome in patients at risk of cardiovascular disease and in patients with diabetes in particular.

#### Allopurinol

Allopurinol is an inhibitor of the enzyme, XO, which catalyzes the conversion of hypoxanthine, via xanthine, to uric acid. As such, allopurinol is an effective treatment for gout, caused by the accumulation of uric acid crystals in the joints. However, allopurinol is also considered to be a XO-specific antioxidant because the enzyme uses molecular oxygen as the electron acceptor during the oxidation process, generating ROS as a by-product. This has relevance to a number of diseases because XO predominantly exists in its dehydrogenase form, which does not generate ROS; conversion of the dehydrogenase to XO can be caused by oxidative modification of the enzyme or irreversible proteolysis. The amount of XO-derived ROS is dependent on the preponderance of XO as well as the availability of substrate (hypoxanthine and xanthine). In heart failure, hypoxanthine and xanthine concentrations increase as a result of cellular damage and there is also the potential for oxidative modification to generate more XO in this disease. As a result, serum uric acid is recognized to be an independent marker of heart failure severity (239) and a possible surrogate marker of oxidative stress in heart failure. Animal studies indicate that allopurinol, among other inhibitors of XO (e.g., oxypurinol), effectively reduce mortality and improve left ventricular function in models of heart failure (240, 241). Some clinical studies using intravenous or intracoronary infusion of XO inhibitors support these findings (242, 243), but the majority of the randomized clinical trials (244–249) have failed to replicate the beneficial findings in animal studies, although there are some hints of improvement in ejection fraction [reviewed in Ref. (250)]. The lack of consistency between the animal and clinical studies is frustrating, but no doubt highlights the complexity of the human disease profile, in which XO-derived ROS are only a contributory element.

## Conclusion

Given that oxidative stress is a key player at various levels in the atherogenic process, it is a reasonable assumption that antioxidant therapy would be an effective therapy in this setting. Indeed, puzzling anomalies in epidemiological data (the French paradox) have been attributed to high-antioxidant ingestion specific to a given population and *in vitro* studies using relatively high concentrations of a wide range of antioxidants support the notion that antioxidants have protective effects. However, associations between plasma concentrations of antioxidant vitamins (A, C, and E) and protection against cardiovascular disease have proved elusive and large intervention trials with these vitamins have failed to conclusively show any benefit. In hindsight, the failure of antioxidant vitamins to show benefit is unsurprising for a range of fundamental reasons that expose several generalizations relating to oxidative stress and antioxidants. First, ROS are not universally harmful; repeated, low-level exposure to ROS is a vital trigger for up-regulation of endogenous antioxidants. Second, redox is a question of balance and the concept of flooding cells with dietary antioxidants to combat oxidative stress is flawed, not only because there are powerful physiological processes in place to ensure that these agents are kept within reasonably strict limits, but also because driving cells into a highly reducing state is likely to be harmful too. Third, many of the diet-derived antioxidants (e.g., polyphenols) are very effectively screened out by the gut or rapidly metabolized and excreted. Plasma concentrations are typically in the nanomolar range – too low to have a direct impact on antioxidant capacity. Paradoxically, it is some of these dietary micronutrients that are perhaps the most promising in terms of cardiovascular outcome, but they should not be considered to be direct antioxidants in this regard. Instead, polyphenolic compounds, or more likely the metabolites that they yield, probably act as mild toxins to drive endogenous defense mechanisms, including up-regulation of a battery of cellular antioxidant systems (168). The irony is that the direct antioxidant activity of polyphenols is almost irrelevant in their protective properties; instead, it is their toxic properties that could prove decisive in determining their efficacy in this regard. Achieving a better understanding of the mechanisms by which polyphenols work is essential in driving the paradigm shift away from the concept that foods high in antioxidants will improve cardiovascular health. Perhaps the focus should be on the diversity and frequency of exposure to dietary polyphenols that should be the focus of public health messages rather than amount.

### Fruit and vegetables vs. supplements

Plants synthesize an enormous variety of chemicals with powerful antioxidant properties, and many clinical studies support the notion that increased consumption of fruit and vegetables have beneficial effects with respect to various markers of cardiovascular disease (84, 251). It is a reasonable extrapolation to suggest that the benefits of fruit and vegetables might be attributable to the most abundant antioxidants that are often present in these foods, namely, vitamins A, C, and E. However, the benefits of vitamin-rich foods have not been matched by large-scale supplement studies, perhaps suggesting that it is not these agents that are responsible to the beneficial effects, but some of the micronutrients that accompany the antioxidant vitamins in these foods. Alternatively, it might be that the whole is greater than the sum of its parts and that there is a synergistic effect of the rich combination of micronutrients and vitamins in fruit and vegetable that bring about the benefit – an effect that is lost in isolated supplements or in multivitamin formulations. Either way, the lack of alignment of results from whole fruit and vegetables compared with vitamin supplements would suggest that vitamins alone do not constitute the answer with respect to antioxidant benefits of certain foods in cardiovascular disease.

### *In vitro* and *in vivo* studies

Why then, the dichotomy between *in vitro* (e.g., cell culture) and small-scale clinical studies compared with larger clinical trials? The answer to this question is vexing, but the reason is likely to lie in the detail of experimental procedures. For example, many *in vitro* experiments use concentrations of antioxidants that are much higher (sometimes orders of magnitude greater) than those that are likely to be found *in vivo* after oral ingestion. Second, it is clear that the digestive system has an enormous impact on the survival or otherwise of antioxidants; for example, large polyphenolic compounds undergo significant hydrolysis and it is widely acknowledged that only small phenolic compounds pass into the plasma. Third, the combination of antioxidants that one might receive from ingestion of fruit and vegetables could be crucial in providing significant benefit – telling in this regard are the data from studies that demonstrate a benefit of elevated vitamin C from increased fruit and vegetable ingestion, but not from vitamin C supplements alone. It is entirely plausible that the plasma vitamin C measured in the fruit and vegetable studies was a marker of the dietary intervention, but a red herring with respect to causality. In addition, there may be other benefits of fruit and vegetables beyond antioxidants; plant-derived sterols, for example, have a modest impact on plasma LDL levels (252). Finally, the end point that is most important in terms of cardiovascular benefit is reduced mortality due to myocardial infarction and stroke. Cardiovascular events are precipitated by plaque rupture, a process that is perhaps influenced by inflammation within the plaque, but does not necessarily correlate with atherosclerotic load. It is quite possible, therefore, that subtle improvements in endothelial function or lipid peroxidation might not have a great deal of impact on the predisposition to plaque rupture.

Despite a wealth of *in vitro* and *in vivo* animal data, the cardiovascular benefits of antioxidant have not yet been proven, particularly with respect to reduced mortality, but there is sufficient evidence in the literature to suggest that there is considerable promise of improving some aspects of cardiovascular health through increased fruit and vegetable consumption. Whether the same is true of isolated antioxidant vitamins, minerals, or polyphenols is less convincing, but as we gain a better understanding of the complexity of the mechanisms involved, there is increased potential to find diet or drug-derived antioxidant agents with beneficial effects, though not necessarily on account of their ability to directly scavenge radicals. Priming the adaptive response on account of low-grade, repeated toxicity, in a process with some parallels to those now thought to be important in underpinning the benefits of exercise, looks like it might hold more promise than attempting to flood the system with antioxidants.

In conclusion, antioxidants should not be considered to be either a panacea or a false hope with respect to cardiovascular disease prevention. To achieve success, however, it is necessary for studies to be carefully designed; the choice of antioxidant should be informed by the target disease, the confirmed role of oxidative stress, and the relevant cellular compartment, as well as the potential antioxidant deficiencies in the target population.

## Conflict of Interest Statement

The authors declare that the research was conducted in the absence of any commercial or financial relationships that could be construed as a potential conflict of interest.
